# Factors Secreted by Cancer-Associated Fibroblasts that Sustain Cancer Stem Properties in Head and Neck Squamous Carcinoma Cells as Potential Therapeutic Targets

**DOI:** 10.3390/cancers10090334

**Published:** 2018-09-17

**Authors:** Saúl Álvarez-Teijeiro, Cristina García-Inclán, M. Ángeles Villaronga, Pedro Casado, Francisco Hermida-Prado, Rocío Granda-Díaz, Juan P. Rodrigo, Fernando Calvo, Nagore del-Río-Ibisate, Alberto Gandarillas, Francisco Morís, Mario Hermsen, Pedro Cutillas, Juana M. García-Pedrero

**Affiliations:** 1 Department of Otolaryngology, Hospital Universitario Central de Asturias and Instituto de Investigación Sanitaria del Principado de Asturias; Instituto Universitario de Oncología del Principado de Asturias, University of Oviedo, 33011 Oviedo, Spain; cristinagarciainclan@gmail.com (C.G.-I.); angelesvillaronga@gmail.com (M.Á.V.); franjhermida@gmail.com (F.H.-P.); rocigd281@gmail.com (R.G.-D.); juanpablo.rodrigo@sespa.es (J.P.R.); nagoredelrio@gmail.com (N.d.-R.-I.); mariohermsen@gmail.com (M.H.); 2 CIBERONC, 28029 Madrid, Spain; 3 Cell Signalling & Proteomics Group, Barts Cancer Institute, Queen Mary University of London, London EC1M 6BQ, UK; p.m.casado-izquierdo@qmul.ac.uk (P.Ca.); p.cutillas@qmul.ac.uk (P.Cu.); 4 Tumour Microenvironment Team, Division of Cancer Biology, Institute of Cancer Research, 237 Fulham Road, London SW3 6JB, UK; fernando.calvo@icr.ac.uk; 5 Cell Cycle, Stem Cell Fate and Cancer Lab Instituto de Investigación Marqués de Valdecilla (IDIVAL), 39011 Santander, Spain; agandarillas@idival.org; 6 EntreChem SL, Vivero Ciencias de la Salud, 33011 Oviedo, Spain; fmv@entrechem.com

**Keywords:** head and neck squamous cell carcinoma, cancer-associated fibroblasts, cancer stem cells, tumor microenvironment, secretome, therapeutic target

## Abstract

This study investigates for the first time the crosstalk between stromal fibroblasts and cancer stem cell (CSC) biology in head and neck squamous cell carcinomas (HNSCC), with the ultimate goal of identifying effective therapeutic targets. The effects of conditioned media from cancer-associated fibroblasts (CAFs) and normal fibroblasts (NFs) on the CSC phenotype were assessed by combining functional and expression analyses in HNSCC-derived cell lines. Further characterization of CAFs and NFs secretomes by mass spectrometry was followed by pharmacologic target inhibition. We demonstrate that factors secreted by CAFs but not NFs, in the absence of serum/supplements, robustly increased anchorage-independent growth, tumorsphere formation, and CSC-marker expression. Modulators of epidermal growth factor receptor (EGFR), insulin-like growth factor receptor (IGFR), and platelet-derived growth factor receptor (PDGFR) activity were identified as paracrine cytokines/factors differentially secreted between CAFs and NFs, in a mass spectrometry analysis. Furthermore, pharmacologic inhibition of EGFR, IGFR, and PDGFR significantly reduced CAF-induced tumorsphere formation and anchorage-independent growth suggesting a role of these receptor tyrosine kinases in sustaining the CSC phenotype. These findings provide novel insights into tumor stroma–CSC communication, and potential therapeutic targets to effectively block the CAF-enhanced CSC niche signaling circuit.

## 1. Introduction

Mounting evidence indicates that tumors are highly complex heterogeneous structures in which growth is supported not only by the cancer cells themselves, but also the surrounding microenvironment. Since cancer must be considered as a systemic disease, an in-depth understanding of tumor progression requires knowledge of the role of both tumor cells and infiltrating stroma, as well as how these distinct cell types interact to drive tumor biology. The tumor stroma is constituted by various types of stromal cells and the extracellular matrix (ECM), collectively denominated as tumor microenvironment (TME) [1].

Fibroblasts are a major component within the TME, and in particular, cancer-associated fibroblasts (CAFs) play a key role in tumorigenesis, as they significantly contribute to important hallmarks necessary for cancer progression, such as sustained growth, invasion, inflammation, angiogenesis, metastasis, and therapeutic resistance [2,3]. It is well established that tumor cells can stimulate stromal cells to release paracrine factors that facilitate cancer growth and dissemination. Specifically, CAFs emerge as critical players in this process, stimulating cancer progression toward aggressive phenotypes through cell–cell communication with cancer cells or other stromal cells, remodeling the ECM and releasing a plethora of growth factors, chemokines, cytokines, and matrix metalloproteinases (MMPs) in the TME [4]. Accordingly, the presence of CAFs has been widely associated with poor prognosis in numerous tumor types, including among others, gastric, colorectal, breast, and prostate cancers [5,6].

Tumors are hierarchically organized with different cancer cell subpopulations, where cancer stem cells (CSCs) are essential for tumor initiation, treatment resistance, relapse, and metastasis [7,8]. CSCs are regulated by, and in turn regulate, cells within the TME. Recent findings have shown the plasticity and phenotype switching of the different cancer cell subpopulations [9,10,11]. Thus, malignant epithelial cells may dedifferentiate, and thereby enter back into the stem cell pool. Therefore, therapies aimed at targeting CSCs within the tumor will not be curative if the CSCs pool can be continuously regenerated from plastic non-CSCs capable of dedifferentiating and reentering the CSC state. There are strong indications that CAFs may regulate CSCs in various ways: (i) acting directly on the CSCs subpopulation to promote their self-renewal; (ii) re-inducing a stem cell phenotype in more differentiated tumor cells (reprogramming); or (iii) activating autocrine signaling loops in tumor cells that maintain them into a stem cell-like state [12]. Consequently, the identification of molecules responsible for the conversion of non-CSCs into CSCs is indispensable to select the most appropriate drugs, or combinations of them, to efficiently eliminate CSCs populations, and subsequently reduce the risk of metastasis outgrowth and tumor relapse.

Previous functional studies have demonstrated that secreted proteins, acting as paracrine factors, provide an important bidirectional communication system between cancer cells and the surrounding fibroblasts. Hence, the secretome from both tumor and stromal cells may constitute a rich reservoir of potential biomarkers and/or new therapeutic targets [13,14]. Likewise, as secreted proteins, they have the potential of being released into blood circulation or saliva, thus increasing the possibility of their detection in patient-derived body fluids.

Therefore, a deeper understanding of the molecules involved in the interaction between CSCs and CAFs is fundamental to find novel targets to block effectively the communication between them, and ultimately prevent their cooperative roles in promoting tumor progression. This study investigated for the first time the crosstalk between stromal fibroblasts and CSC in the context of head and neck squamous cell carcinomas (HNSCC). Using mass spectrometry (MS) we identified various CAF-secreted molecules potentially responsible for sustaining CSC properties in HNSCC-derived cell lines. In addition, pharmacological targeting of signaling pathways related to the identified factors effectively blocked CAF-induced CSC phenotype, thus suggesting their potential as novel therapeutic targets to overcome CSC-mediated disease progression and resistance to therapy.

## 2. Results

### 2.1. Fibroblast-Secreted Factors Sustain Cancer Stem Properties of HNSCC Cells

**Figure 1:**
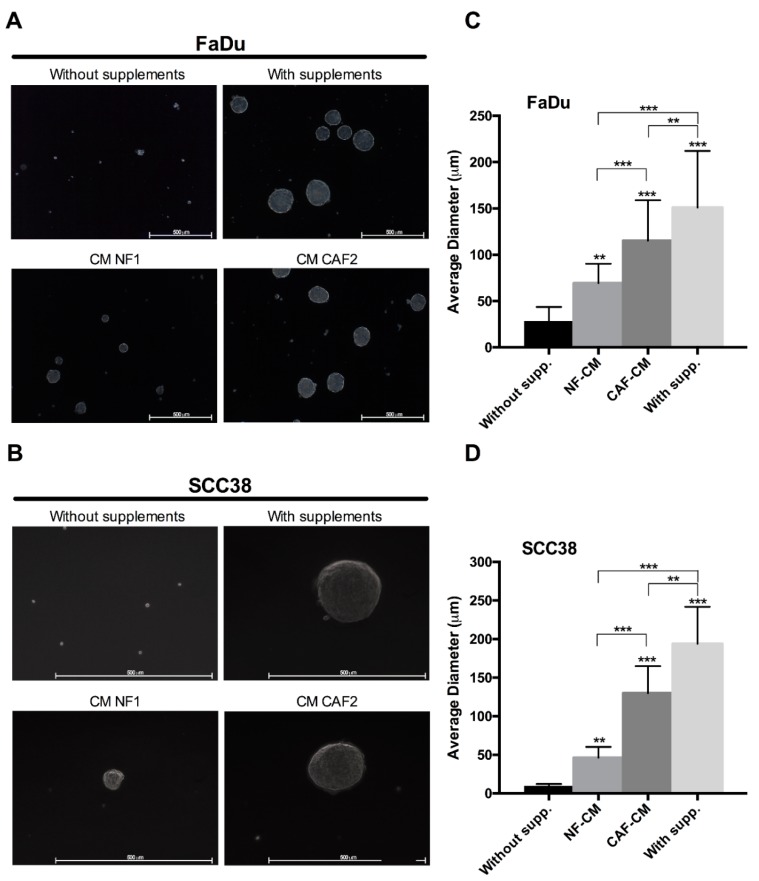
Effect of fibroblast-CM (conditioned media) on the tumorsphere formation capacity of head and neck squamous cell carcinomas (HNSCC) cells. Representative images of orospheres formed by (**A**) FaDu and (**B**) SCC38 cells in non-supplemented medium, supplemented medium, and CM from normal fibroblasts (NFs) or cancer-associated fibroblasts (CAFs). Bar chart showing the average diameter of spheroids formed by (**C**) FaDu and (**D**) SCC38 cells in the previous conditions. All data were expressed as the mean ± SD of at least three independent experiments performed. Scale bar: 500 μm. *** *p* < 0.001 and ** *p* < 0.01 by Holm-Sidak’s multiple comparisons test.

Cancer stem cells (CSCs) play critical roles in tumor initiation, progression, recurrence, and treatment resistance. However, the crosstalk between CAFs and CSCs in the context of HNSCC has not yet been explored. This prompted us to investigate the effect of CM from CAFs or NFs on cancer stem properties in HNSCC cells, such as tumorosphere formation, anchorage-independent growth, and expression of CSC markers. We first assayed the ability of fibroblast-CM to promote the formation of clonal spheres (tumorspheres) in non-adherent and serum-free culture conditions (Figure 1). We found that both FaDu and SCC38 cells were able to form orospheres when grown in NF-CM, and much more efficiently in CAF-CM, showing bigger size spheres similar to those formed in the presence of stem supplemented medium, used as a positive control by its high efficiency to induce orosphere formation. As expected, HNSCC cells were unable to form orospheres in non-supplemented medium, which served as a negative control.

**Figure 2:**
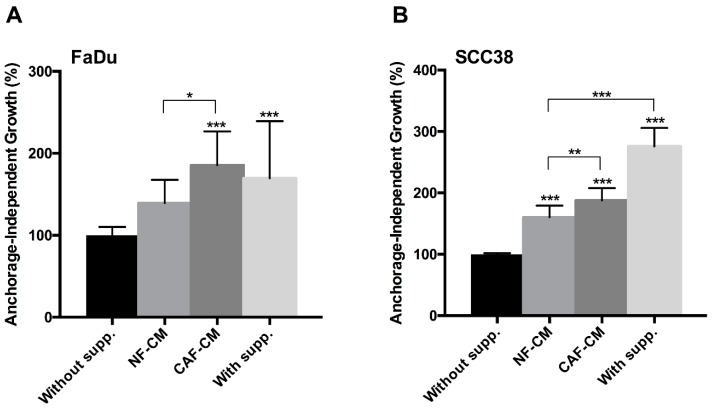
Effect of fibroblast-CM on anchorage-independent growth of HNSCC cells. (**A**) FaDu and (**B**) SCC38 cells were seeded in plates coated with Poly(2-hydroxyethyl methacrylate) (polyHEMA) and grown in non-supplemented medium, CM from NFs, CM from CAFs, or supplemented medium. Cell proliferation was estimated by tetrazolium-based MTS assay after 4 days. Data were normalized to the absorbance at day 0 and relative to control (non-supplemented) cells. All data were expressed as the mean ± SD of at least three independent experiments performed in quadruplicate. *** *p* < 0.001, ** *p* < 0.01 and * *p* < 0.05 by Holm-Sidak’s multiple comparisons test.

Consistently, we also found that CAF-CM, and to a less extent NF-CM, significantly increased anchorage-independent growth in both FaDu and SCC38 cells, compared to non-supplemented medium (Figure 2). These results indicate that components of the CAF-CM promote CSC properties in HNSCC cells.

### 2.2. Fibroblast-Secreted Factors Induced the Expression of Stemness-Related Genes in HNSCC Cells

The results above suggest that CAF-CM may be inducing stem properties in HNSCC cells. To verify this observation and to gain further insight into the mechanisms by which stromal fibroblasts may promote CSC features in HNSCC cells, we analyzed the expression of several CSC markers and other genes with well-known functions in pluripotency, self-renewal, and signal transduction in CSCs.

**Figure 3:**
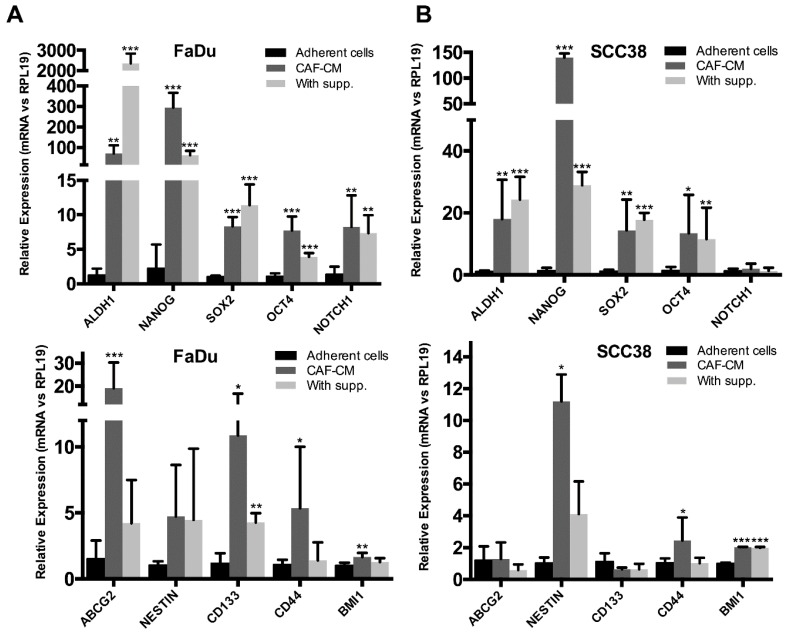
Effect of fibroblast-CM on the expression of stem-related genes in HNSCC cells. Bar chart showing the expression analysis of CSC-related genes by qRT-PCR analysis in FaDu (**A**) and SCC38 (**B**) orospheres formed in CAF-CM and supplemented medium. Adherent monolayer cultures of FaDu or SCC38 cells were used as control. Data were normalized to RPL19 levels and relative to control cells. All data were expressed as the mean ± SD of at least three independent experiments performed in triplicate. * *p* < 0.05, ** *p* < 0.01 and *** *p* < 0.001 by Student’s *t*-test.

Thus, we performed RT-qPCR in orospheres formed by FaDu and SCC38 cells treated with CAF-CM or supplemented medium and gene expression was compared to that of adherent control cultures. Overall, we observed an increase in the mRNA levels of several CSC-related genes in orospheres formed in CAF-CM and supplemented medium, compared to adherent cells (Figure 3). Thus, CAF-CM potently and consistently increased the mRNA levels of ALDH1, NANOG, SOX2, and OCT4. There were some noticeable differences between supplemented medium and fibroblast-CM-formed orospheres, depending on the gene and also the HNSCC-derived cell line. ALDH1 expression levels were highly induced in orospheres formed in supplemented medium in FaDu cells, whereas mRNA levels of NANOG, ABCG2, CD44, CD133, and Nestin were more robustly induced in CAF-CM-formed orospheres in HNSCC cells. These results suggest that CAF-CM and supplemented medium regulate stemness by activating different signal transduction programs in HNSCC cells to sustain the CSC-phenotype.

### 2.3. Identification of Fibroblast-Secreted Proteins by Mass Spectrometry

We next performed a Mass Spec analysis in NFs and CAFs to identify the repertoire of CAF-secreted proteins that may be responsible for sustaining cancer stem properties in HNSCC cells. Comparison of protein expression levels in the secretomes was conducted using three different biological replicates for each cell line.

**Figure 4:**
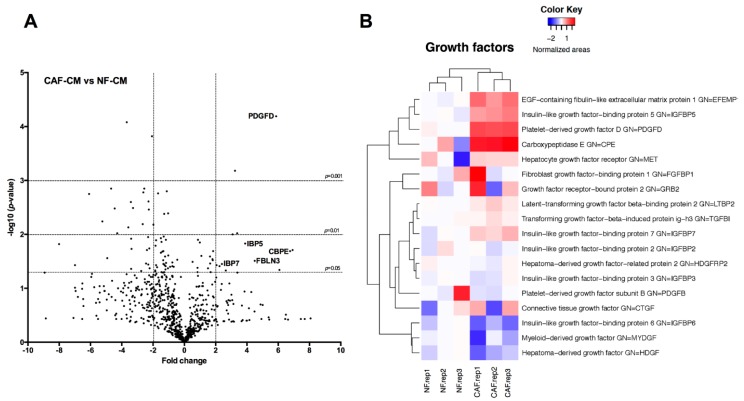
Mass Spec analysis of extracellular proteins differentially secreted by CAFs versus NFs. (**A**) Volcano plot showing the global secretome changes, illustrating fold change (log base 2) and *p*-value (−log base 10), between CAFs and NFs. Horizontal bars represent the significance *p* = 0.05, *p* = 0.01 and *p* = 0.001 (proteins under horizontal bar of *p* = 0.05 did not reach significance). Vertical bars represent the proteins with a fold change higher than 2 or −2; (**B**) Heatmap represents the changes in the growth factors related-proteins found in the secretome. Three independent experiments are shown; red indicates fold changes >0 and blue indicates fold changes <0.

Secretome analysis provided valuable information on the proteins differentially secreted by CAFs compared to NFs (Figure 4). We identified 41 extracellular proteins differentially secreted by CAFs versus NFs (fold change > 2 or <−2) (Table 1). Among the most highly up-regulated proteins, we selected for further study Carboxypeptidase E (CBPE), which has been implicated in cell proliferation and survival in other cancer types [15,16], as well as platelet-derived growth factor D (PDGFD), epidermal growth factor (EGF)-containing fibulin-like extracellular matrix protein-1 (FBLN3), insulin-like growth factor binding protein-5 (IBP5) and insulin-like growth factor binding protein-7 (IBP7), respectively associated to growth factor-signaling pathways through PDGF, EGF, and insulin-like growth (IGF) receptors [17,18,19].

### 2.4. Targeting EGFR, IGFR, and PDGFR Signaling Effectively Inhibited CAF-Promoted Stemness in HNSCC Cells

We hypothesized that the CAF-secreted proteins CBPE, PDFGD, FBLN3, IBP5, and IBP7 may enhance CSC properties in HNSCC cells through the activation of signaling pathways involving the activity of CBPE or receptors for EGF, IGF, and PDGF. In order to support this hypothesis, we assessed the ability of CAF-CM to induce anchorage-independent growth in the presence of specific pharmacologic inhibitors, as these are conveniently available as research tools. We used drugs whose modes of action involve both receptor blockage (Gefitinib for EGFR, OSI-906 for IGFR, and CP-673451 for PDFGR) and protein blockage (GEMSA for CBPE) [16,17,18,19]. In addition, we also include the mithramycin analog EC-8042, since it has been recently described as a potent inhibitor of stemness-related genes and CSCs viability in other cancers [20].

**Figure 5:**
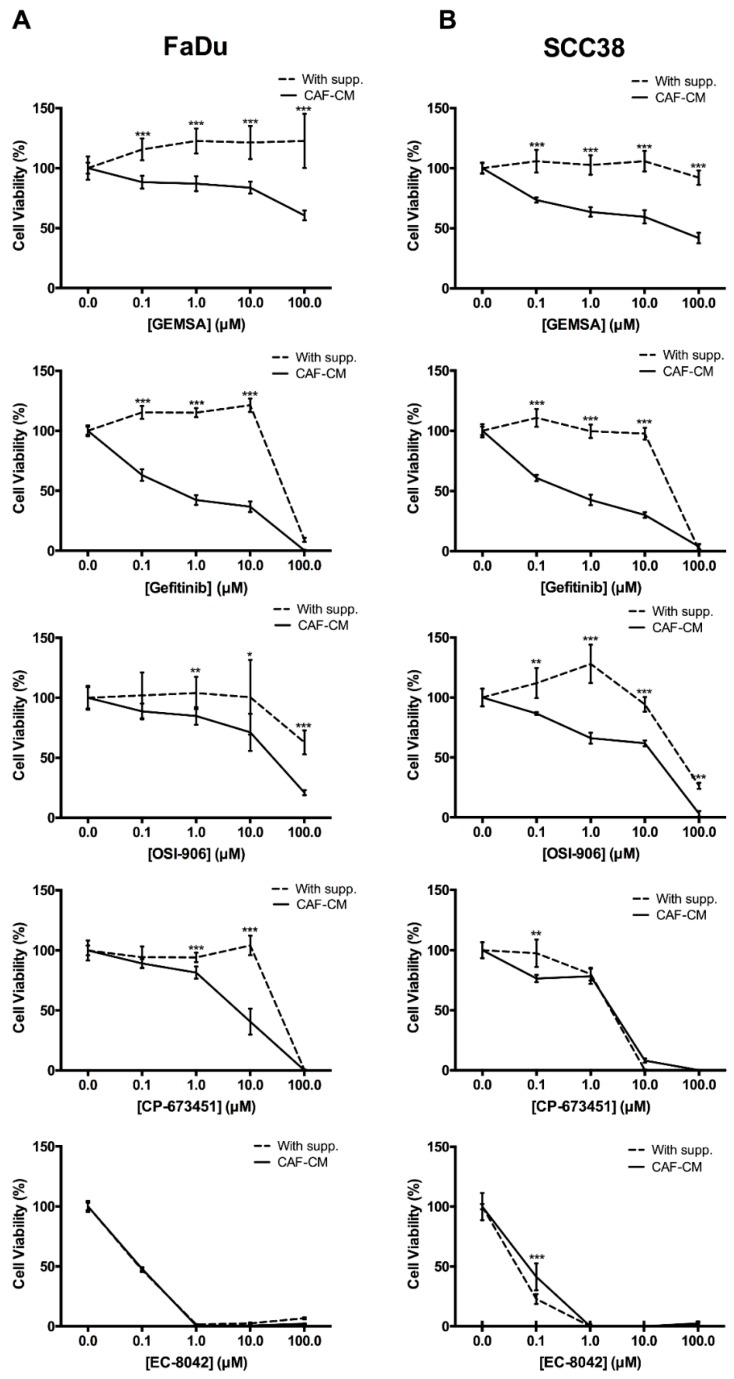
Effect of 2-guanidinoethylmercaptosuccinic acid (GEMSA), Gefitinib, OSI-906, CP-673451, and EC-8042 on CAF-CM-mediated anchorage-independent growth. (**A**) FaDu and (**B**) SCC38 cells were seeded in polyHEMA-coated plates and grown in CAF-CM or supplemented medium. After 24 h, cells were treated with increasing concentrations of the indicated drugs (GEMSA, Gefitinib, OSI-906, CP-673451, and EC-8042). Cell proliferation was estimated by tetrazolium-based MTS assay after 4 days. Data were normalized to the absorbance at day 0 and relative to control (vehicle-treated) cells. All data were expressed as the mean ± SD of at least three independent experiments performed in quadruplicate. * *p* < 0.05, ** *p* < 0.01 and *** *p* < 0.001 by Student’s *t*-test.

Results showed that GEMSA, Gefitinib, OSI-906, and CP-673451 reduced anchorage-independent growth in a dose-dependent manner in both FaDu and SCC38 cells grown in CAF-CM (Figure 5). However, cells grown in supplemented-medium were clearly less sensitive to these drugs, with only high doses having cytotoxic effects. EC-8042 was an effective blocker of anchorage-independent growth in both FaDu and SCC38 cells grown in either CAF-CM or supplemented-medium.

**Figure 6:**
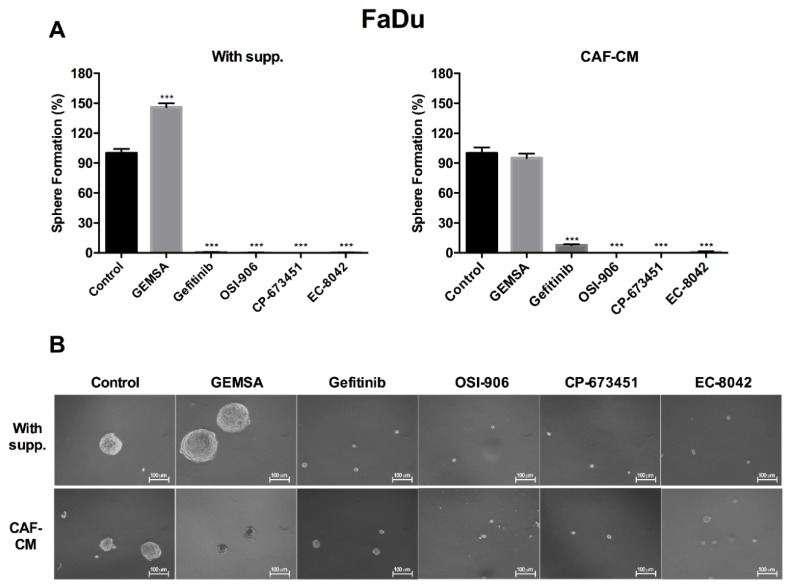
Effect of GEMSA, Gefitinib, OSI-906, CP-673451, and EC-8042 on FaDu orosphere formation. (**A**) Bar chart showing orospheres formation ability of FaDu grown in supplemented medium (left) or CAF-CM (right) and treated with GEMSA (10 μM), Gefitinib (1 μM), OSI-906 (10 μM), CP-673451 (5 μM), and EC-8042 (0.01 μM); (**B**) Representative images of FaDu orospheres for each condition shown in the bar chart. Sphere formation was estimated by tetrazolium-based MTS assay after 10–12 days. All data were expressed as the mean ± SD of at least three independent experiments performed in quadruplicate. *** *p* < 0.001 by Student’s *t*-test. Scale bar: 100 μm.

We next evaluated the effect of these compounds on orosphere formation. Our previous results (Figure 5) provided us with information on the most appropriate concentration of compounds to be used for these experiments. Thus, we found that Gefitinib (1 μM), OSI-906 (10 μM), CP-673451 (5 μM), and EC-8042 (0.01 μM) effectively prevented tumorsphere-forming capability of FaDu cells in either CAF-CM or supplemented medium (Figure 6). In contrast, 10 μM GEMSA did not significantly inhibit orosphere formation in CAF-CM, whilst increased orosphere formation in supplemented medium. Together, these results suggest that modulators of EGFR, PDGF and IGFR activity and also the mithramycin analog EC-8042 have the potential to inhibit stemness-related properties in HNSCC cells, consequently emerging as potential therapeutic targets to effectively block the CAF-enhanced CSC niche signaling circuit.

## 3. Discussion

Recent increasing evidence has suggested that the TME is an integral and inseparable part of malignant transformation [21,22], as it plays a significant role during tumor progression, enabling primary growth, invasion, and metastatic spreading [23,24,25]. Hence, the study of the role of the different TME components and strategies aimed at interfering with the crosstalk between cancer cells and their cellular partners in the TME is of great interest, since it may provide novel promising anti-cancer therapies with minimal chance to develop drug resistance [26].

This study provides original evidence demonstrating that CAF-secreted factors sustain and robustly enhance stemness in HNSCC-derived cell lines, thereby increasing anchorage-independent growth, tumorsphere formation, and expression of various CSC markers, such as NANOG, SOX2, OCT4, ALDH1, CD133, CD44, and NOTCH1, in the absence of serum or any other supplements. There are strong indications that CAFs regulate CSCs [12]. In this regard, Donnarumma and colleagues observed that CAFs promoted cancer progression by enhancing stemness, Epithelial-mesenchymal transition (EMT) phenotype, and anchorage-independent growth in breast cancer [27]. Also, CAFs were found to secrete ADAM10-rich exosomes to promote cell motility and activate RhoA and Notch signaling in several cancer cell lines [28]. Vermeulen et al. showed that primary colon CAFs released HGF to induce nuclear translocation of ß-catenin in tumor cells and a stem cell-like transcription profile [29]. In prostate cancer, tumor cells released IL-6 leading to fibroblasts activation, and in turn, fibroblasts, through MMPs secretion, elicited an EMT phenotype in cancer cells, as well as enhancement of tumor growth and development of spontaneous metastases. CAF-induced EMT in prostate carcinoma cells was accompanied by increased expression of CSC markers, and enhanced ability to form tumorspheres and self-renewal [30].

Nevertheless, prior to this study, the crosstalk between CAFs and CSC in the context of head and neck squamous cell carcinomas (HNSCC) had not been explored. To identify the molecules responsible for mediating the conversion of non-CSCs into CSCs is indispensable to select the most appropriate drugs, or combinations of them, to efficiently eliminate CSCs populations, and subsequently reduce the risk of metastasis outgrowth and tumor relapse.

These reasons prompted us to perform an unbiased proteomic analysis using MS to identify the proteins secreted to extracellular media by stromal fibroblasts that mediate paracrine communication. Our secretome analysis showed that there were several differentially secreted proteins (over- or under-expressed) in CAFs compared with NFs. Among the most promising and highly induced factors that could be responsible for sustaining the CSC phenotype are FBLN3, IBP5, IBP7, and PDGFD.

FBLN3 is an extracellular protein that can bind to EGFR, inducing EGFR autophosphorylation and the activation of downstream signaling pathways. Several groups have observed that EGFR is over-expressed in a wide spectrum of tumors, including HNSCC [31,32]. EGFR overexpression results in aggressive tumor behavior, radiation resistance, and poor prognosis [33]. EGFR activates several downstream pathways, including Ras/Raf/MAPK/ERK, PI3K/Akt, STAT, and the PLC-γ signaling pathways to potentiate growth and survival of tumor cells and CSCs [19].

IBP5 and IBP7, as IGF binding proteins, bind IGFs to regulate their activity by prolonging their half-life and circulation turnover, and by controlling their binding to IGFR, either positively or negatively, affecting the IGF signaling pathway [34]. IBPs may inhibit mitogenesis, differentiation, survival, and other IGF-stimulated events by sequestering IGFs away from the IGFR [35,36]. Also, they can function independently of IGF signaling pathway via interacting with proteins other than IGFs binding their own membrane receptors [37]. IBP5 has recently been deemed a molecular biomarker for predicting response to therapy and clinical outcome in patients with different cancers [17]. Also, it has been implicated as a cancer promoter or cancer repressor in various tumor types to regulate migration, differentiation, cell attachment, and cell morphology [38,39]. Although there is less evidence for the role of IBP7 in cancer, some groups have reported that IBP7 can promote cancer progression. In non-small cell lung carcinoma, IBP7 was associated with metastatic disease [40]. In T-cell acute lymphoblastic leukemia, IBP7 inhibited proliferation by causing G0/G1 arrest and induced drug resistance [41]. In gastric cancer, IBP7 overexpression was associated with tumor progression and poor survival [42].

PDGFD has recently gained tremendous amount of attention due to its involvement in carcinogenesis. In agreement with the oncogenic function of PDGFD in human malignancies, PDGFD overexpression has been detected in a variety of cancers including prostate, lung, renal, ovarian, brain, and pancreatic cancer [18,43]. PDGFD regulates multitude cellular pathways including PI3K/Akt, NF-κB, Notch, ERK, mTOR, MAPK, VEGF, MMPs, Cyclin D1 and BCL2 [44,45,46,47,48,49]. Moreover, PDGFD has been found to regulate the EMT process that is important for tumor metastasis [44,45,50,51]. Prostate cancer cells with an EMT phenotype induced by PDGFD displayed CSC features, including increased expression of SOX2, NANOG, OCT4, and Notch-1, and enhanced sphere-forming ability and rapid tumor growth in vivo [44]. In addition, it has also been reported that tissue-resident stem cells induce EMT through interaction with the TME via PDGFD, thereby leading to increased number of CSCs and tumor growth [50].

Since FBLN3, IBP5, IBP7, and PDGFD play oncogenic roles through the regulation of tumor cell growth, invasion, metastasis, EMT, and CSCs, targeting these proteins, their receptors, or downstream signaling pathways could be valuable strategies to interfere with stromal-cancer cell heterotypic communication. Interestingly, we found that pharmacologic inhibition of EGFR, IGFR, and PDGFR signaling pathways efficiently blocked CAF-induced orosphere formation and anchorage-independent growth in HNSCC cells. Nevertheless, it is plausible that the potent anti-stemness effects observed by targeting EGFR, IGFR, and PDGFR pathways could also be due to a direct effect on the tumor cells, since these signaling pathways are frequently altered in different cancers, including HNSCC. A limitation of this study is that only one population of primary CAFs and NFs were used.

## 4. Materials and Methods

### 4.1. Drugs

EC-8042 (EntreChem, Oviedo, Spain), OSI-906 (Selleckchem, Suffolk, UK), CP-673451 (Selleckchem, Suffolk, UK), 2-Guanidinoethylmercaptosuccinic acid (GEMSA) (Abcam, Cambridge, UK), and Gefitinib (TOCRIS Bioscience, Bristol, UK) were prepared as 1 mM solutions in sterile DMSO or water, according to manufacturer’s indications, for in vitro experiments, maintained at −20 °C and brought to the final concentration just before use.

### 4.2. Cell Culture

FaDu cells were purchased to the American Type Culture Collection, and the HNSCC cell line SCC38 derived from a primary tumor (T2N0M0) was kindly provided by Dr. R. Grenman (Department of Otolaryngology, University Central Hospital, Turku, Finland) [52]. Primary cancer-associated fibroblasts (CAFs) were obtained from minced tumor tissue of surgically resected HNSCC at the Hospital Universitario Central de Asturias. Normal dermal fibroblasts (NF) were obtained from the dermis of human neonatal foreskin, by enzymatic cell disaggregation as described [53]. Cell line authentication was performed by DNA (STR) profiling at the SCT Core Facilities (University of Oviedo, Asturias, Spain). All cell lines were tested periodically for mycoplasma contamination by PCR to specifically amplify a conserved region of the mycoplasma 16S ribosomal RNA gene (Biotools Detection kit, Madrid, Spain).

HNSCC cells and fibroblasts were grown in DMEM (Biowest, Nuaillé, France) supplemented with 10% fetal bovine serum (FBS) (Gibco, Waltham, MA, USA), 100 U/mL penicillin (Biowest, Nuaillé, France), 200 mg/mL streptomycin (Biowest, Nuaillé, France), 2 mM L-glutamine, 20 mM HEPES (pH 7.3) (Biowest, Nuaillé, France), and 100 mM MEM non-essential amino acids (Biowest, Nuaillé, France).

### 4.3. Conditioned Media Production

Primary CAFs and NFs were grown in T175 flask (Corning, Corning, NY, USA) with complete medium until reaching 80–90% confluence (approx 4–5×10^6^ cells). Then, medium was replaced and cells were grown for 72 h in 20 mL of DMEM-F12 (GE Healthcare, Pittsburg, PA, USA) with 100 U/mL penicillin (Biowest, Nuaillé, France), 200 mg/mL streptomycin (Biowest, Nuaillé, France) in the absence of supplements or FBS and also without phenol red (for Mass Spec analysis). Next, conditioned media (CM) were collected and filtered through a 0.45 μm pore filter (Sigma-Aldrich, St. Louis, MO, USA) and frozen at −80 °C until use.

### 4.4. Anchorage-Independent Cell Growth

For anchorage-independent cell growth, normal 96-well tissue culture plates (Corning) were coated with 10 g/L of the anti-adhesive polymer poly-2-hydroxyethyl methacrylate (polyHEMA, Sigma-Aldrich) in 95% ethanol and dried at 56 °C for 16 h to prevent cell attachment. PolyHEMA-coated plates were sterilized with UV-light for 30 min before use.

FaDu and SCC38 cells were plated into 96-well tissue culture plates at a density of 10,000 cells per well. Cell proliferation was measured after 4 days. Quantification of cell number was determined in quadruplicates using a tetrazolium-based MTS test (CellTiter 96 Aqueous One Solution Cell Proliferation Assay from Promega, Madison, WI, USA), reading the absorbance at 490 nm with the use of a Synergy HT plate reader (BioTek, Winooski, VT, USA).

### 4.5. Orosphere Formation Assay

HNSCC-derived cells lines were plated at a density of 500 cells/mL in 75-cm^2^ flask (5000 cells) or 6-well tissue culture plates (1000 cells/well), treated with a sterile solution of polyHEMA (10 g/L in 95% ethanol) (Sigma-Aldrich) to prevent cell attachment, and grown in either DMEM-F12 (GE Healthcare, Pittsburgh, PA, USA) without any supplements, conditioned media from CAFs or NFs, or the standard cancer stem cell medium [54,55], which is composed of DMEM-F12 supplemented with 1% Glutamax (Life Technologies, Waltham, MA, USA), 2% B27 Supplement (Life Technologies, Waltham, MA, USA), 20 ng/mL human EGF (PeproTech, London, UK), 10 ng/mL human bFGF (PeproTech, London, UK), 4 µg/mL insulin (Sigma-Aldrich), 100 U/mL penicillin, and 200 mg/mL streptomycin. The above medium will be referred to in this manuscript as supplemented medium as opposed to non-supplemented DMEM-F12 medium.

After 10–12 days, well-formed spheres were photographed in Leica Microsystems microscope DMIL T, coupled with a Leica DC500 High-resolution Digital Camera (Leica Microsystems, Barcelona, Spain). Then the spheres were centrifuged at 500 rpm for 5 min, washed with PBS and collected for RNA extraction or MTS assay.

### 4.6. RNA Extraction and Real-Time RT-PCR 

Total RNA was extracted from HNSCC cells using Trizol reagent (Invitrogen Life Technologies, Waltham, MA, USA), and cDNA synthesized with Superscript II RT-PCR System (Invitrogen Life Technologies), according to manufacturer’s protocols. Gene expression was analyzed by Real-time PCR using the StepOnePlus Real-Time PCR System (Applied Biosystems, Waltham, MA, USA) following Applied Biosystems’ SYBR Green Master Mix protocol. Reactions were carried out using the primers detailed in Table S1. The constitutively expressed RPL19 ribosomal coding gene was used as endogenous control. The relative mRNA expression was calculated using the 2^−ΔΔCT^ method.

### 4.7. Secretome Analysis by Mass Spectrometry

Conditioned media from NFs and CAFs were obtained as above described, and protein concentration was determined by Pierce BCA Protein Assay Kit (Thermo Fisher, Waltham, MA, USA). Three independent batches of CM were produced from each experimental condition and used as biological replicates.

50 μg of protein (for each CM) were digested overnight with immobilized TLCK-trypsin (20 TAME units/mg) (Thermo Scientific, Waltham, MA, USA) for 16 h at 37 °C. The resultant peptide solutions were desalted by solid phase extraction (SPE) using Oasis HLB extraction cartridges (Waters UK Ltd., Manchester, UK) according to manufacturer’s instructions. Briefly, cartridges coupled to a vacuum manifold set at 5 mm Hg, were activated with 1 mL of 100% acetonitrile (ACN) and equilibrated with 1.5 mL of wash solution (1% ACN, 0.1% TFA in water). After the cartridges were loaded with peptide solution, they were washed with 1 mL of wash solution. Peptides were eluted with 0.5 mL of 50% ACN containing 0.1% TFA and dried in a speed vacuum centrifuge.

Peptide pellets were solubilized in 0.1% TFA and run in two mass spectrometry platforms consisting in an LTQ-Orbitrap XL (Thermo Scientific, Waltham, MA, USA) coupled to a nanoACQUITY ultra performance LC (Waters Corp., Milford, MA, USA) and a QExactive plus (Thermo Scientific) online connected to a Ultimate 3000 RSLC chromatographer (Thermo Scientific). For the LTQ-Orbitrap XL system, peptides were loaded into a nanoACQUITY trap column and separated on BEH C18 nano ACQUITY column. Separation was performed using a 180-min gradient with solvent B 5–25% at a flow rate of 300 nL/min (mobile phase B; 100% ACN and 0.1% FA; mobile phase A; 100% water and 0.1% FA). The mass spectrometer was operated in data-dependent acquisition mode for top 5 CID acquisitions. For the QExactive plus system, peptides were loaded into an Acclaim PepMap 100 trap column and separated on an Acclaim PepMap RSLC analytical column. Separation was performed using a 120-min gradient with solvent B 5–25% at a flow rate of 300 nL/min (mobile phase B; 100% ACN and 0.1% FA; mobile phase A; 100% water and 0.1% FA). The mass spectrometer was operated in data-dependent acquisition mode for top 15 CID acquisitions.

### 4.8. Mass Spectrometry Data Analysis

Raw files were converted to peak lists (in the Mascot Generic Format) using Mascot Distiller (version 2.3.0) and searched using Mascot Server (version 2.3.01) against the SwissProt Uniprot database (2012-03-10) restricted to the relevant taxonomy. Mass windows for tolerance for MS scans were 10 ppm and 600 mmu for MS/MS. Fixed modification of carbamidomethylation of cysteine and variable modifications of oxidation of methionine and glutamine to pyroglutamate conversion were permitted. Mascot result files were parsed using a Perl script that uses the Mascot Parser files provided by Matrix Science. The threshold for accepting peptides as being positively identified was set at an expectancy score of 0.05.

For individual peptide quantification, we used Pescal, a software that automates the construction of extracted ion chromatograms for all peptides identified across all samples being compared [56,57]. Independent Pescal analyses were run for the quantification of peptides identified in the LTQ-Orbitrap XL or QExactive systems. Protein intensity calculation was automated with an in house developed script that sums the signals of peptides obtained from both platforms and comprised in the same protein. Data were normalized by dividing each protein intensity by the sum of all protein intensities within a sample. Fold changes were calculated by averaging the normalized intensities of peptides within a sample group and dividing these by the intensities of the control group. Fold changes were then log transformed before calculation of significance using an unpaired *t*-test.

### 4.9. Statistical Analysis

The data are presented as the mean ± standard deviation (SD), unless otherwise stated, and compared using unpaired Student’s *t*-test or one-way ANOVA test and Holm-Sidak’s multiple comparisons test. The normality of the data was analyzed using the Kolmogorov-Smirnov test. Statistical analysis was performed using GraphPad Prism version 6.0 (GraphPad Software Inc., La Jolla, CA, USA). *p* values less than 0.05 were considered statistically significant (* *p* < 0.05; ** *p* < 0.01; *** *p* < 0.005).

## 5. Conclusions

Together our findings uncover novel insights into the tumor stroma–CSC communication, and provide also a novel therapeutic rationale to effectively block the CAF-enhanced CSC niche signaling circuit, to ultimately overcome CSC-mediated disease progression and resistance to therapy.

## Figures and Tables

**Table 1:** Proteins differentially secreted in CAFs versus NFs.

UNIPROT_ID	Gene Name	Fold Change CAFs	*p*-Value
HNRPL_HUMAN	heterogeneous nuclear ribonucleoprotein L(HNRNPL)	**6.92**	**0.020**
**CBPE_HUMAN**	**carboxypeptidase E(CPE)**	**6.76**	**0.020**
CO7_HUMAN	complement C7(C7)	**6.08**	**0.046**
**PDGFD_HUMAN**	**platelet derived growth factor D(PDGFD)**	**5.87**	**>0.001**
**FBLN3_HUMAN**	**EGF containing fibulin like extracellular matrix protein 1(EFEMP1)**	**4.50**	**0.031**
**IBP5_HUMAN**	**insulin like growth factor binding protein 5(IGFBP5)**	**3.89**	**0.015**
DDAH1_HUMAN	dimethylarginine dimethylaminohydrolase 1(DDAH1)	**3.38**	**0.010**
PGM1_HUMAN	phosphoglucomutase 1(PGM1)	**3.24**	**0.001**
GREM1_HUMAN	gremlin 1, DAN family BMP antagonist(GREM1)	**3.09**	**0.010**
IF4A1_HUMAN	eukaryotic translation initiation factor 4A1(EIF4A1)	**2.65**	**0.047**
RS18_HUMAN	ribosomal protein S18(RPS18)	**2.40**	**0.036**
TCPQ_HUMAN	chaperonin containing TCP1 subunit 8(CCT8)	**2.25**	**0.039**
**IBP7_HUMAN**	**insulin like growth factor binding protein 7(IGFBP7)**	**2.06**	**0.037**
TCPD_HUMAN	chaperonin containing TCP1 subunit 4(CCT4)	**−2.07**	**>0.001**
MARCS_HUMAN	myristoylated alanine rich protein kinase C substrate(MARCKS)	**−2.22**	**0.042**
ANXA2_HUMAN	annexin A2(ANXA2)	**−2.24**	**0.018**
PEDF_HUMAN	serpin family F member 1(SERPINF1)	**−2.27**	**0.003**
MMP3_HUMAN	matrix metallopeptidase 3(MMP3)	**−2.29**	**0.002**
PSA5_HUMAN	proteasome subunit alpha 5(PSMA5)	**−2.31**	**0.037**
CALR_HUMAN	calreticulin(CALR)	**−2.32**	**0.011**
BASP1_HUMAN	brain abundant membrane attached signal protein 1(BASP1)	**−2.39**	**0.020**
VASN_HUMAN	vasorin(VASN)	**−2.45**	**0.034**
LUM_HUMAN	lumican(LUM)	**−2.56**	**0.001**
CFAD_HUMAN	complement factor D(CFD)	**−2.62**	**0.002**
LA_HUMAN	Sjogren syndrome antigen B(SSB)	**−2.66**	**0.007**
UB2V1_HUMAN	TMEM189-UBE2V1 readthrough(TMEM189-UBE2V1)	**−2.76**	**0.048**
PSG7_HUMAN	pregnancy specific beta-1-glycoprotein 7 (gene/pseudogene)(PSG7)	**−3.28**	**0.003**
PTGDS_HUMAN	prostaglandin D2 synthase(PTGDS)	**−3.38**	**0.019**
FBLN2_HUMAN	fibulin 2(FBLN2)	**−3.40**	**0.012**
AN32B_HUMAN	acidic nuclear phosphoprotein 32 family member B(ANP32B)	**−3.40**	**0.044**
ENPP2_HUMAN	ectonucleotide pyrophosphatase/phosphodiesterase 2(ENPP2)	**−3.42**	**0.003**
MASP1_HUMAN	mannan binding lectin serine peptidase 1(MASP1)	**−3.58**	**0.007**
EMIL2_HUMAN	elastin microfibril interfacer 2(EMILIN2)	**−3.68**	**>0.001**
CSPG4_HUMAN	chondroitin sulfate proteoglycan 4(CSPG4)	**−4.29**	**0.010**
APOE_HUMAN	apolipoprotein E(APOE)	**−4.45**	**0.003**
TENA_HUMAN	tenascin C(TNC)	**−4.68**	**0.001**
PDIA6_HUMAN	protein disulfide isomerase family A member 6(PDIA6)	**−4.93**	**0.028**
A2GL_HUMAN	leucine rich alpha-2-glycoprotein 1(LRG1)	**−5.24**	**0.006**
RAB2A_HUMAN	RAB2A, member RAS oncogene family(RAB2A)	**−6.09**	**0.002**
RL15_HUMAN	ribosomal protein L15(RPL15)	**−6.99**	**0.037**
PSG4_HUMAN	pregnancy specific beta-1-glycoprotein 4(PSG4)	**−8.01**	**0.015**

Up-regulated proteins are shown in red and down-regulated proteins in blue.

## Supplementary Materials

The following are available online at http://www.mdpi.com/2072-6694/10/9/334/s1, Supplementary Table S1: Primers used for real-time RT-PCR.

## Abbreviations

CAFs	Cancer-associated fibroblasts
CSC	Cancer stem cells
HNSCC	Head and neck squamous cell carcinomas
EMT	Epithelial-mesenchymal transition
NFs	Normal fibroblasts
EGF	Epidermal growth factor
EGFR	Epidermal growth factor receptor
IGF	Insulin-like growth factor
IGFR	Insulin-like growth factor receptor
PDGF	Platelet-derived growth factor
PDGFR	Platelet-derived growth factor receptor
ECM	Extracellular matrix
TME	Tumor microenvironment
MMPs	Matrix metalloproteinases
MS	Mass spectrometry
GEMSA	2-Guanidinoethylmercaptosuccinic acid
FBS	Fetal bovine serum
HEPES	4-(2-hydroxyethyl)-1-piperazineethanesulfonic acid
CM	Conditioned media
polyHEMA	poly-2-hydroxyethyl methacrylate
FGF	Fibroblast growth factor
SPE	Solid phase extraction
CAN	Acetonitrile
TFA	Trifluoroacetic acid
FA	Formic acid
CBPE	Carboxypeptidase E
PDGFD	Platelet-derived growth factor D
FBLN3	EGF-containing fibulin-like extracellular matrix protein-1
IBPs	Insulin-like growth factor binding protein
IBP5	Insulin-like growth factor binding protein-5
IBP7	Insulin-like growth factor binding protein-7
HGF	Hepatocyte growth factor
IL-6	Interleukin-6
