# Supplementary material for: Factors Secreted by Cancer-Associated Fibroblasts that Sustain Cancer Stem Properties in Head and Neck Squamous Carcinoma Cells as Potential Therapeutic Targets
Source: Cancers (Basel). 2018 Sep 17;10(9):334. doi: 10.3390/cancers10090334 (PMC6162704; doi:10.3390/cancers10090334)
Supplement: Supplementary file 1 [file cancers-10-00334-s001.pdf]

Supplementary materials: Factors Secreted by Cancer-Associated Fibroblasts that Sustain Cancer Stem Properties in Head and Neck Squamous Carcinoma Cells as Potential Therapeutic Targets

Saúl Álvarez-Teijeiro, Cristina García-Inclán, M. Ángeles Villaronga, Pedro Casado, Francisco Hermida-Prado, Rocío Granda-Díaz, Juan P. Rodrigo, Fernando Calvo, Nagore del-Río-Ibisate, Alberto Gandarillas, Francisco Morís, Mario Hermsen, Pedro Cutillas and Juana M. García-Pedrero

Table S1. Primers used for real-time RT-PCR (5′→3′).

Genes	Forward	Reverse
ABCG2	ATGGATTTACGGCTTTGCAG	TCTTCGCCAGTACATGTTGC
ALDH1	AACAGTGTGGGTGAATTGCT	GGAAACCGTACTCTCCCAGT
BMI1	CTGGAGAAGGAATGGTCCAC	CACTTTCCAGTTCTCCAGCA
CD133	CAGTGAGAAAGTGGCATCGT	TCCTATGCCAAACCAAAACA
CD44	GATCATCTTGGCATCCCTCT	TGAGTCCACTTGGCTTTCTG
NANOG	TGCTTATTCAGGACAGCCCT	TCTGGTCTTCTGTTTCTTGACT
NESTIN	CAGCGTTGGAACAGAGGTTG	GCTGGCACAGGTGTCTCAAG
NOTCH1	AGACGGCATCAACACGGCCTTC	GTGTAGCTGTCCACGCAGTCCG
OCT4	GGCAACCTGGAGAATTTGTT	ACTCGGACCACATCCTTCTC
RPL19	GCGGAAGGGTACAGCCAAT	GCAGCCGGCGCAAA
SOX2	TCAGGAGTTGTCAAGGCAGAGAAG	CTCAGTCCTAGTCTTAAAGAGGCAGC
